# Morphologic and cytomorphometric analysis of exfoliated buccal mucosal cells in diabetes patients

**DOI:** 10.4103/0970-9371.73291

**Published:** 2010-10

**Authors:** H Prasad, V Ramesh, PD Balamurali

**Affiliations:** Department of Oral Pathology and Microbiology, Modern Dental College and Research Centre, Indore, India; 1 Department of Oral Pathology and Microbiology, Mahatma Gandhi Post Graduate Institute of Dental Sciences, Pondicherry, India

**Keywords:** Oral exfoliative cytology, cytomorphometry, diabetes, nuclear size, glycosylated hemoglobin

## Abstract

**Background:**

It is now known that the disease process of diabetes has effects on various tissues of the body. The following study was done to analyze the effects of diabetes on oral tissues.

**Aims:**

To study the morphology and cytomorphometry of the cells obtained in cytologic smears from the buccal mucosa of diabetic patients.

**Materials and Methods:**

Smears were obtained from clinically normal buccal mucosa of 50 randomly selected diabetic patients attending the diabetic clinic and the out-patient department and of five healthy subjects as control. Smears were stained using Papanicolaou method, and using a micrometer mean values of nuclear diameter (ND), cell diameter (CD), cytoplasmic diameter (CyD) and nucleus: cytoplasm ratio (N: C ratio) were obtained for each patient. Diabetic patients were divided into four groups based on the glycosylated hemoglobin (GHb) values for comparison.

**Statistical analysis used:**

Student’s *T*-test and Fisher’s *F*-test.

**Results:**

Statistically significant increase in ND (*P*=0.0367) was found in diabetic patients compared to controls. Degree of glycemic control significantly affected ND (*P*=0.0042) and N: C ratio (*P*=0.0055). In general, as the severity of diabetes increases, ND and N: C ratio rise gradually.

**Conclusions:**

Diabetes produces definite morphologic and cytomorphometric changes in the buccal mucosa of patients. However, further research in this direction is indicated, to analyze the significance of these findings as a tool for diabetes detection, as well as to obtain deeper insights into its effects on various tissues.

## Introduction

Diabetes mellitus is a growing and massive silent epidemic that has the potential to cripple health services in all parts of the world.[1] It is basically characterized by chronic hyperglycemia, associated with disturbances in the metabolism of carbohydrates, proteins and lipids, as a result of absolute or relative insulin deficiency. Chronic hyperglycemia causes damage to the eyes, kidneys, nerves, heart and blood vessels. The etiology and pathophysiology leading to the hyperglycemia, however, are markedly different among patients with diabetes mellitus, dictating different prevention strategies, diagnostic screening methods and treatments.[2]

Diabetes often goes undiagnosed because many of its symptoms seem so harmless. Some common symptoms include frequent urination, excessive thirst and hunger, unusual weight loss, increased fatigue and blurry vision. Worldwide, millions of people are affected with diabetes and the number is climbing steeply. Currently, a diagnosis of diabetes is achieved by evaluating the blood glucose levels. Either a random blood sugar estimation or analysis of fasting/post-prandial blood sugar levels is the commonest diagnostic test for diabetes. Recently, however, monitoring of glycosylated hemoglobin (GHb) levels has become much commoner. Estimation of GHb level is not affected by factors like diet or medication intake, and it gives an accurate and objective measure of glycemic control over the past 3 months.[3] Hence GHb estimation is preferred by many to monitor diabetic patients.

The disease process of diabetes results in many problems of dental interest. Several studies suggest a higher prevalence and severity of some pathologies in the oral tissues of diabetic patients, like gingivitis, periodontitis, candidiasis and other opportunistic infections.[4] It has been shown that diabetes may also cause various changes in the cells of the oral mucosa, which can be determined by exfoliative cytology.[5] Application of quantitative techniques has largely improved the potential accuracy of exfoliative cytology.[6] Therefore, a study of cytomorphometry in oral exfoliative cytology was taken up to assess the usefulness of this procedure in the diagnosis and follow-up of diabetes patients. The knowledge of this procedure, especially with regard to the morphology and morphometry of the cells, may enable its use as another diagnostic tool of simplified nature, for diabetes mellitus.

## Materials and Methods

Patients attending the diabetic clinic and the outpatient department of our institute, were included in this study. Informed consent was obtained from each individual and a data sheet was completed, detailing the name, age, sex, relevant medical history, etc. Only patients with a known history of diabetes at least for the past 6 months were included in the study group. Patients were included irrespective of whether they were under any medications for diabetes or not. Control group included normal healthy adult individuals with no history of diabetes or any other illnesses. Biochemical measurements (i) to rule out anemia (defined as hemoglobin concentration less than 9 gm%) and (ii) to determine the GHb concentration were done in all the subjects included in the study. Patients with habits like tobacco or alcohol intake; those with anemia or any other systemic illnesses or those who were under any medications other than for diabetes were excluded, because previous studies have shown that cell and nuclear sizes are influenced by these factors.[7–11]

A total of 50 diabetic patients and 5 control subjects were included in the study. Diabetic patients were also grouped into the following four categories for further analysis based on their GHb levels, which indicates the degree of glycemic control achieved:

- Well-controlled diabetics (WCD) - GHb ≤8%
- Moderately controlled diabetics (MCD) - GHb >8% and ≤10%
- Poorly controlled diabetics (PCD) - GHb >10% and ≤12%
- Uncontrolled diabetics (UCD) - GHb >12%

Smears were taken from clinically normal buccal mucosa of the patients using a wooden spatula moistened in distilled water. The scrapings were then transferred to clean glass slides previously marked with the patient’s reference number, and spread thinly and uniformly with a circular motion over the middle third of the slide. The smears were immediately fixed in 95% ethanol and stained by the Papanicolaou method.

Fifty clearly defined cells were measured in each case using an eyepiece micrometer to obtain the nuclear diameter (ND) and cell diameter (CD), Cytoplasmic diameter (CyD) and nucleus: cytoplasm ratio (N:C) values were then derived using simple mathematical calculations. The data were then analysed using statistical methods (Student’s *T*-test and *F*-test).

## Results

Statistical analysis of the data obtained showed that the ND and N:C ratio of cells from diabetic patients was significantly higher than cells from control subjects.

Table 1 shows the mean data for the control group and the study group. This shows that the ND is significantly greater (*P*=0.0367) in the study group than in the control group. The N:C ratio is also similarly increased, though not at a significant level.

**Table 1:** Comparison of study parameters between the experimental and control groups

Parameter	Group	*N*	Mean	SD	*t*-value	*P* value
Nuclear diameter	Control	5	9.00	0.37	–2.14	0.0367
	Experimental	50	9.57	0.59		
Cell diameter	Control	5	54.97	3.09	0.33	0.7437
	Experimental	50	54.32	4.32		
N:C ratio	Control	5	0.197	0.015	–1.60	0.1164
	Experimental	50	0.216	0.027		
Cytoplasmic diameter	Control	5	45.97	3.06	0.62	0.5398
	Experimental	50	44.74	4.33		

N:C = Nucleus:Cytoplasm

Table 2 shows the mean data for the various groups of diabetic patients. It was found that the level of diabetic control, as noted by the GHb values, definitely influenced ND (*P*=0.0042) and N:C ratio significantly (*P*=0.0055). CD and CyD were also influenced, but not at a significant level. These findings show that the severity of diabetes (or in other words, the amount of glycemic control) affects all the four study parameters.

**Table 2:** Influence of glycemic control on the four study parameters

Parameter	Group	*N*	Mean	SD	*F*-value	*P* value
N.D	Well controlled	11	9.17	0.33	5.05	0.0042
	Moderately controlled	15	9.44	0.68		
	Poorly controlled	17	9.78	0.43		
	Uncontrolled	7	10.01	0.60		
C.D	Well controlled	11	54.22	2.47	1.84	0.1527
	Moderately controlled	15	54.96	5.04		
	Poorly controlled	17	55.19	4.32		
	Uncontrolled	7	50.96	4.10		
N:C ratio	Well controlled	11	0.204	0.017	4.79	0.0055
	Moderately controlled	15	0.210	0.030		
	Poorly controlled	17	0.217	0.022		
	Uncontrolled	7	0.246	0.023		
Cy.D	Well controlled	11	45.06	2.603	2.27	0.0934
	Moderately controlled	15	45.52	5.043		
	Poorly controlled	17	45.41	4.248		
	Uncontrolled	7	40.95	3.857		

**Figure 1:**
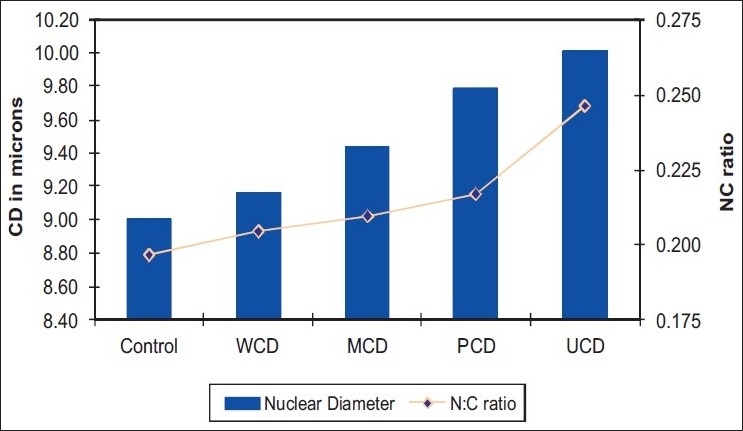
Mean nuclear diameter (ND) and nucleus: cytoplasm (N:C) ratio values

Table 3 shows the comparison of ND and N:C ratio between control group and the various groups of diabetic patients [Figure 1]. It is seen from the table that the value of ND rises steadily from the control group to the uncontrolled diabetic group. ND in the UCD group is raised significantly when it is compared with the other groups. The N:C ratio is found to follow a similar trend as the ND. It is raised significantly in the UCD group when it is compared with the other groups.

**Table 3:** Comparison of ND and N:C ratio between control and various groups of diabetes

Parameter	Control	WCD	MCD	PCD	UCD
ND (in *µ*)	9.00	9.17	9.44	9.78	10.01
N:C Ratio	0.197	0.204	0.210	0.217	0.246

**Figure 2:**
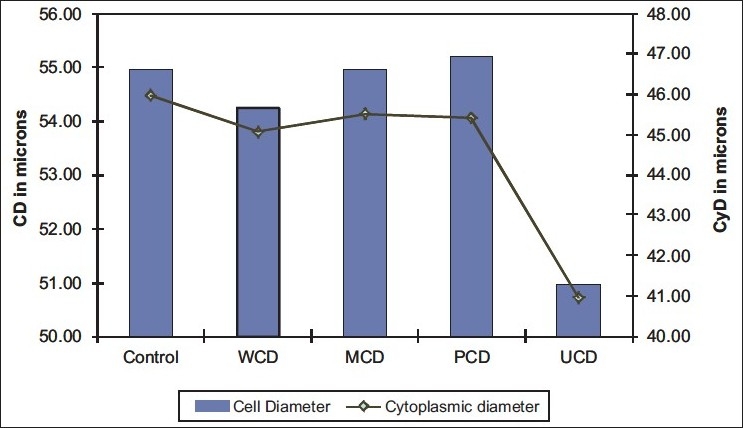
Mean cell diameter (CD) and cytoplasmic diameter (CyD) values in various groups

CD and CyD [Table 4] were similarly compared between the various groups, and it was found that both these parameters again showed similar trends. Both CD and CyD gradually increase in the MCD and PCD groups, but in the UCD group there was a sudden decrease in both [Figure 2].

**Table 4:** Comparison of CD and CyD between control and various groups of diabetes

Parameter	Control	WCD	MCD	PCD	UCD
CD (in *µ*)	54.97	54.22	54.96	55.19	50.96
CyD (in *µ*)	45.97	45.06	45.52	45.41	40.95

Age and gender of the patients were found to have no bearing on the morphometric changes of the cells. Similarly, the duration of diabetes (as determined from the patient’s history) was found to have no influence on the study parameters. However, this was not statistically analyzed because duration of diabetes is a subjective entity; it cannot be exactly determined when a patient will start having diabetes.

**Figure 3:**
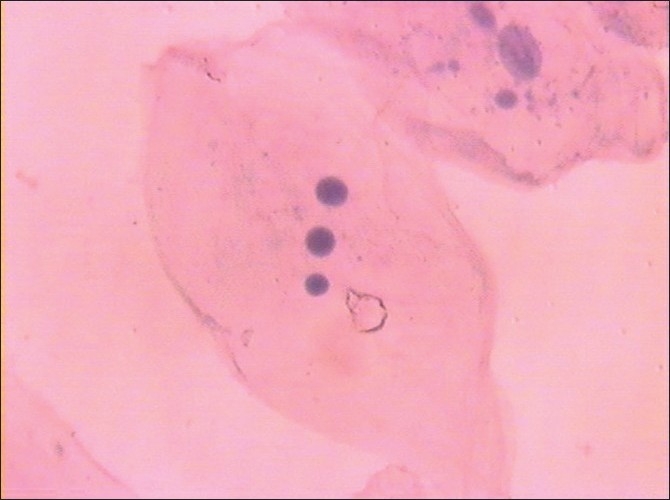
Abnormal cytoplasmic inclusions (Pap, ×100)

**Figure 4:**
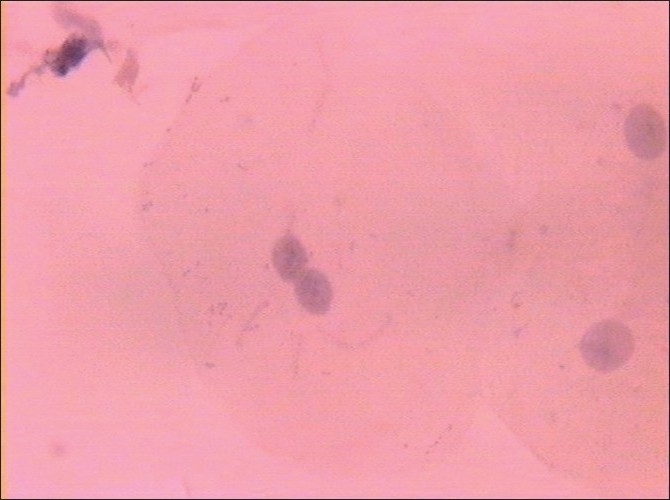
Binucleated cell (Pap, ×100)

**Figure 5:**
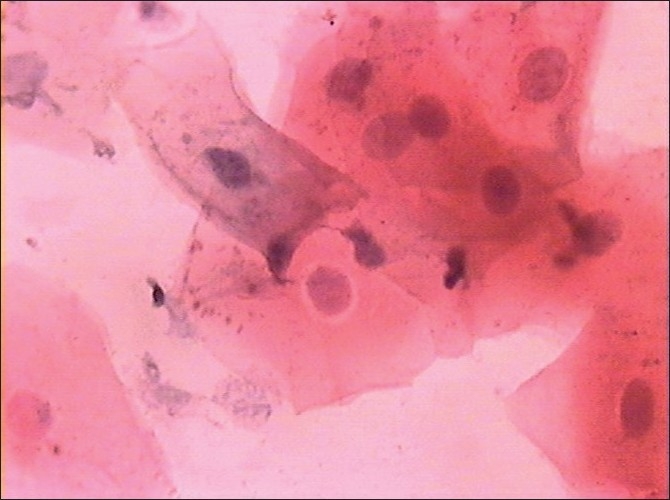
Perinuclear halos (Pap, ×100)

**Figure 6:**
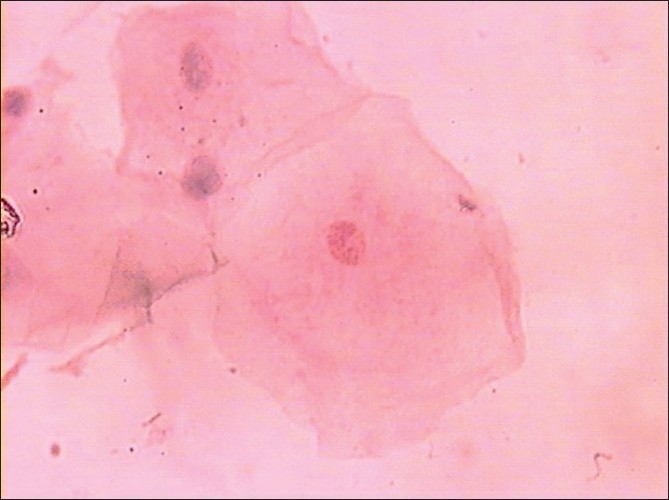
Karyorrhexis (Pap, ×100)

Morphological changes were also noted in smears obtained from diabetic patients, but these were not subjected to any statistical analysis. These changes were in the form of binucleation, karyorrhexis, micronuclei, perinuclear halos, cytoplasmic inclusions and vacuolizations, etc [Figures 3–6].

## Discussion

In our present study, we have measured the ND, CD, CyD and N:C ratio of exfoliated buccal mucosa cells using a micrometer in the diabetic patients (*n*=50) and control subjects (*n*=5). The purpose of our study is to determine if any significant differences in these parameters are present in diabetic individuals, and if this can be used as a diagnostic criterion for diabetes.

The diabetic patients were grouped under various grades of glycemic control, based on their GHb levels. Analysis of the four study parameters between these groups showed that glycemic control definitely influenced ND and N:C ratio. To further clarify the influence of glycemic control on the various study parameters, each parameter was individually compared across control group and various grades of diabetes. The ND showed a consistent and uniform increase in diameter from control to uncontrolled diabetic group. This increase in ND is highly significant (99%) in poorly controlled and uncontrolled diabetic patients. This finding concurs with the study by Alberti *et al*.,[5] who report a significant increase in nuclear area in diabetic patients. However, they have not taken the degree of glycemic control into account.

Increase in nuclear size might be an indicator of cellular ageing in diabetic patients. Decreased cellular turnover as a result of ischemia following atherosclerosis would result in more number of mature cells with large nuclei in the smear. However, this has to be confirmed by studying the oral epithelial turnover rate in diabetic patients. Ageing would also produce various morphologic alterations in cells in the form of pleomorphism, bilobed nuclei, cytoplasmic vacuolizations, etc.[12] We were able to see such morphologic variations in cells obtained from diabetic patients.

Diabetic patients also suffer from dehydration and this (when combined with the decreased salivary flow rates) may lead to mucosal atrophy. When cytologic samples from atrophic mucosa is made, it is possible that more of basal and parabasal cells may get included, thus leading to an increased ND.[10,11]

Patients with diabetes are more likely to have xerostomia and atrophic oral mucosa due to dehydration caused by the disease process as well as due to reported decrease in salivary flow rates. This is also associated with superadded infections like candidiasis, which can evoke a chronic inflammatory response in the oral mucous membrane resulting in an increase in the ND in the basal and parabasal cells and a corresponding decrease in CD and CyD.

The CD and CyD showed almost similar patterns when compared across various groups. There is a significant decrease in CD and CyD in uncontrolled diabetics, compared to other groups. This finding is contradictory to the findings of Jajarm *et al*.[13] who reported an increase in cytoplasmic area in diabetic patients and Alberti *et al*.[5] who concluded that cell area did not show any significant difference in diabetic individuals. Ogden *et al*.[10,11] have reported a decrease in CD in patients with alcoholism, and they have proposed that this might be due to the dehydration seen in them. A similar condition of dehydration is also found in diabetic patients, and this might explain the decrease in CD.

The N:C ratio was also compared across the various groups of diabetes. A gradual increase in N:C ratio is noticed as we progress from control group to poorly controlled diabetics. This is followed by a sudden steep rise in N:C ratio in the uncontrolled diabetics, which is highly significant. This is to be expected because, as we have seen earlier, uncontrolled diabetics show the maximum increase in ND and the maximum fall in CyD.

In summary, the present study was undertaken to analyze the changes in cytomorphometry and morphology in exfoliated buccal mucosa cells in diabetic patients. The following findings were observed:

- There is a clear and definite increase in ND as we progress from normal individuals to patients with uncontrolled diabetes.
- In contrast to the increase in ND, there is a decrease in CD and CyD in uncontrolled diabetes.
- The N:C ratio gradually and steadily increases from normal individuals to uncontrolled diabetics.
- In addition to these morphometric changes, certain morphological changes in the form of binucleation, intracytoplasmic inclusions, micronuclei, perinuclear halo and keratinized squames were also noticed in smears from diabetic patients.

From the present study, we conclude that diabetes produces definite morphological and morphometric changes in the exfoliated buccal mucosal cells. The change in ND is particularly significant. However, for establishing exfoliative cytology as a diagnostic tool for diabetes, further studies with a larger sample size need to be done.
